# Colorado Agricultural Workers' Rights of Access to Key Healthcare Providers: A Policy Brief

**DOI:** 10.3389/fpubh.2022.856878

**Published:** 2022-05-26

**Authors:** Lorann Stallones, Morgan Valley, Whitney Pennington

**Affiliations:** ^1^Department of Psychology, Colorado State University, Fort Collins, CO, United States; ^2^Department of Environmental and Radiological Health Sciences, Colorado State University, Fort Collins, CO, United States

**Keywords:** agriculture, workers, healthcare, access, policy

## Abstract

Health is a human right. In order to exercise their right to health, agricultural workers need to have access to medical, dental, and behavioral health care. Agricultural workers need to have medical, dental, and behavioral health care available at times and locations that are amenable to their work schedules and worksites. Many agricultural workers do not have access to transportation due to the nature of their working conditions, therefore policies to provide transportation will increase access to medical, dental, and behavioral health services.

## Introduction

Agricultural workers experience numerous barriers to realizing their human right to health. Although currently limited, national and state policies provide a path to address and overcome the barriers to accessing medical care for agricultural workers and improve their wellbeing. This brief discusses a state law passed in Colorado in June 2021 that addresses several agricultural labor rights issues (1). We focus on one section of the bill (Section 8-13.5-202) that aims to reduce barriers agricultural workers experience when accessing key service providers including health care providers, community health workers, and promotoras. In addition to summarizing how this section of the bill that may impact agricultural worker health related to access to key service providers, we recommend actions needed to effectively implement the healthcare access issues addressed in the bill. As an immediate first step, we call on the Agricultural Work Advisory Committee established by the bill to build monitoring systems before the proposed rules are implemented. By collecting baseline data and subsequent comparative data, the state can assess the impact of new requirements for access to healthcare, transportation, and communications on agricultural worker health and wellbeing.

## Agricultural Worker Health Risks and Hazards

Agricultural workers are low-paid, often uninsured employees in a hazardous industry. In 2015-2016, the median annual income for a farmworker in the United States ranged from $17,500-$19,999 (2). Specifically, they have a high risk of work-related injuries, occupational skin diseases, eye injuries and exposure to irritants, oral health problems, exposure to sexually transmitted infections, depression, anxiety, obesity, high serum cholesterol and high blood pressure (3). Seasonal and migrant workers are an especially under-served and under-resourced population within the agricultural sector (4). A growing body of research demonstrates the heightened burden of health and safety issues among migrant and seasonal farmworkers in the United States stemming from their exposure to environmental, occupational, and social hazards (5–10). These burdens may be even more pronounced among women migrant workers (11). The COVID-19 pandemic exacerbated inequalities and had a disproportionately negative impact on agricultural workers' health, while causing community-level suffering, stress, and economic challenges (12–14).

In addition to exposure to hazards, agricultural workers also face numerous barriers to accessing proper medical and dental services. These barriers include lack of documentation or work authorization, low formal educational attainment, inadequate transportation, financial strains, lack of medical insurance, lack of documentation, lack of health insurance, limited medical, dental and health care facilities, and lack of culturally and linguistically appropriate services (5, 15). A significant barrier to accessing medical care reported by agricultural workers is the prohibitive cost of care, but workers also report language barriers, transportation difficulties, and distance from services as barriers to accessing needed health care (2). These barriers grew during the COVID-19 pandemic, which led to reduced access to healthcare services including testing and vaccinations (12–14).

While the World Health Organization has identified health as a fundamental human right (16) and academics and labor rights advocates have increasingly called for a human rights-based approach to farmworker health (17), less research has been conducted related to available and accessible health care for agricultural workers. To realize the right to health, agricultural workers must be able to access medical, dental, and behavioral health services. Importantly, agricultural workers need to have medical, dental, and behavioral health care available at times and locations that are amenable to their work schedules and worksites.

## Summary: 2021 Colorado Agricultural Workers' Rights Bill

Introduced in early 2021, the bill titled “Agricultural Workers' Rights” extends numerous worker protections that apply to other industries to agriculture. The bill includes minimum wage, overtime standards, and provision of rest and meal periods; establishes heat illness and injury protection rules, including the provision of water, shade, and annual training; adds responsibilities for agricultural employers during a public health emergency; and guarantees workers access to key service providers.

In this brief, we focus on Bill Section 8-13.5-202, which defines agricultural workers' access to key service providers, including health care providers. Table 1 summarizes the aspects of the bill in Section 8-13.5-202 that address access to health care services and provides our recommended actions to implement the access issues addressed.

**Table 1:** Policies and authors' recommended actions, Colorado (1), Section 8-13.5-202.

**Policy**	**Summary**	**Authors' recommended actions**
Access to health care providers	Specifies times when employers cannot interfere with workers' rights to reasonable access to key providers; phone or internet access during breaks or at employer provided housing	• Establish baseline health care utilization among key providers through a partnership with the Ag Worker Access Campaign • Define adequate phone/internet access on farms, ranches, and at employer provided housing
Access to transportation	Agricultural employees living in employer provided housing will have employer-provided transportation to key providers	• Ensure safe transportation for eligible agricultural workers
Communication regarding agricultural worker rights	Employers will post notice of agricultural workers rights	• Develop template for the information to be posted on workers' rights • Ensure posting is available in appropriate language for specific workers

The first section of the bill mandates access to key providers when the workers are present at the employer-provided housing and prohibits employer interference with these visits. The next prohibits employer interference with agricultural workers' access to key service providers at any location during any time when workers are not involved in paid work or during paid or unpaid rest and meal breaks, and with respect to health-care providers during any time, whether or not they are working. The next section directs the Director of the Colorado Division of Labor Standards and Statistics in the Department of Labor and Employment to promulgate rules regarding additional times during which an employer may not interfere with an agricultural worker's reasonable access to key service providers, including periods during which the agricultural worker is performing compensable work, especially during periods when the agricultural worker is required to work in excess of 40 h per week and may have difficulty accessing such services outside of work hours.

The bill also includes a section in which an employer can require visitors accessing a worksite to follow protocols designed to manage biohazards and other risks of contamination, to promote food safety, and to reduce the risk of injuries to or from livestock on farms and ranches except on open range, if the same protocols are generally applied to any other third parties who may have occasion to enter the worksite. Further, the bill addresses transportation to services for those agricultural employers who provide housing and transportation for workers in which they would provide transportation at least 1 day per week for workers to access basic necessities, conduct financial transactions, and meet with key service providers, including health care providers. This transportation requirement differs for range workers, with transportation required 1 day every three weeks. For agricultural workers with their own vehicles who are permitted to park on the employer properties, the employer is not required to provide transportation. Finally, only agricultural workers will be allowed to bar people from their residences.

Agricultural employers must post notices of the agricultural workers' rights in conspicuous locations, using a variety of communication approaches to ensure workers are aware of their rights under this section of the bill.

Section 8-13.5-205 establishes an agricultural work advisory committee which “shall gather and analyze data and other information regarding the wages and working conditions of agricultural workers and [...] each January 1 [...] shall report its progress, findings, and legislative recommendations” (pages 15-16) to several committees of the Colorado legislature. The law specifically defines the makeup of this nine-member committee: two people who have worked as agricultural workers, two worker's rights advocates, three agricultural employers, and two representatives from Colorado Legal Services' Migrant Farmworker Division. Below we present actionable recommendations for this advisory committee to operationalize in its assessment of the impact of the law. We suggest the advisory committee seeks input and support from state and local agencies, US Centers for Agricultural Safety and Health, other researchers, and migrant healthcare providers in acquiring the data and feedback required to achieve its goals.

## Actionable Recommendations

To assess the success of this policy change, we recommend that the advisory committee establish monitoring systems before the proposed rules are implemented. Currently there is limited information about health care utilization among the agricultural and range workers who might be impacted by the proposed bill. Prior to May 2022, it would be prudent to assess the baseline utilization of medical, dental, and behavioral health care services among these workers. This might be accomplished through partnering with the Ag Worker Access Campaign, which is a partnership between the National Center for Farmworker Health and the National Association of Community Health Centers (http://www.ncfh.org/ag-worker-access.html). The program is designed to increase agricultural worker access to health care. Partnering with this campaign would provide the information needed to determine if the policy has increased access to service providers, as planned.

The issue of transportation should also be reviewed to ensure safe and available transportation can be provided to workers housed by employers. Specifically, determining what type of vehicles will be used and what safety equipment is in place on those vehicles (e.g., seatbelts) and the safety record of drivers of the transportation vehicles is important to ensure workers are not being put into hazardous situations.

The bill also calls for allowing key providers access to the workplaces of this vulnerable group of workers. To comply, employers may need to develop plans which describe how that access can be granted without disruption to other mandated food and biosecurity protocols. Technical assistance and templates could be provided to guide employers in developing a site-specific plan based on the commodities they produce.

Another important issue that should be addressed before May 2022 is to map the key services that are available in the geographic areas where employers and workers are located to be able to develop the materials informing workers not only of their rights with regard to accessing services, but also to provide workers with information about services that are available. While the bill requires informing workers of their rights, there is no mention of employer responsibilities to be familiar with key service providers in their community. Further, there is no mention in the bill about informing key service providers in the community. Therefore, a gap may exist between the intention implied within the bill and the implementation.

Section 14 of the bill included the appropriation of over $700,000 for the 2021-2022 fiscal year (July 1, 2021 – June 30, 2022). The majority of these funds were appropriated to the department of labor and employment to the department of agriculture for the creation and implementation of new standards as promulgated by the bill. Should funds remain from those appropriations, we recommend the departments consider using those funds to address our recommendations above, utilizing the feedback and experience of the bill-established agricultural worker advisory committee to do so.

## Discussion

The Colorado Agricultural Workers' Rights Bill (1) has the potential to improve access to medical, dental, and behavioral health services for agricultural workers. This access is critical to addressing existing health disparities and burdens exacerbated by Covid-19 and mitigating emerging threats to agricultural worker health. The broad policy could impact all agricultural workers in Colorado and may lead to improvements among underserved subgroups including seasonal, migrant, and female workers. If successful, this policy can serve as a model for other states and for the federal agencies that oversee the health and safety of agricultural workers. However, without developing a robust system for monitoring and evaluating the implementation of the policies addressed by the bill, it will remain unknown if this approach improves access to essential services and ultimately takes a step toward realizing health as a right for all agricultural workers in Colorado.

## Author Contributions

LS prepared the initial manuscript. MV and WP reviewed the initial draft, provided edits, and additional sections to the manuscript. All authors contributed to the article and approved the submitted version.

## Funding

High Plains Intermountain Center for Agricultural Health and Safety with a grant from HHS-CDC-Centers for Disease Control and Prevention, National Institute of Occupational Safety and Health (Grant # U54 OH008085) partially supported the manuscript. The views expressed in this manuscript solely reflect the authors and do not represent an official position by HHS/CDC/NIOSH.

## Conflict of Interest

The authors declare that the research was conducted in the absence of any commercial or financial relationships that could be construed as a potential conflict of interest.

## Publisher's Note

All claims expressed in this article are solely those of the authors and do not necessarily represent those of their affiliated organizations, or those of the publisher, the editors and the reviewers. Any product that may be evaluated in this article, or claim that may be made by its manufacturer, is not guaranteed or endorsed by the publisher.
